# Computational and Pharmacological Studies on the Antioxidant, Thrombolytic, Anti-Inflammatory, and Analgesic Activity of *Molineria capitulata*

**DOI:** 10.3390/cimb43020035

**Published:** 2021-06-22

**Authors:** Mohammad Ashiqur Rahman Bhuiyan Shovo, Marzia Rahman Tona, Jannatul Mouah, Fayza Islam, Md. Helal Uddin Chowdhury, Tuhin Das, Arkajyoti Paul, Duygu Ağagündüz, Md. Masudur Rahman, Talha Bin Emran, Raffaele Capasso, Jesus Simal-Gandara

**Affiliations:** 1 Department of Pharmacy, International Islamic University Chittagong, Chittagong 4318, Bangladesh; arshuvo796@gmail.com (M.A.R.B.S.); marziaeva96@gmail.com (M.R.T.); mawajannatul72@gmail.com (J.M.); masud@iiuc.ac.bd (M.M.R.); 2 Department of Pharmacy, Stamford University Bangladesh, Dhaka 1217, Bangladesh; fayzaislammunia@gmail.com; 3 Ethnobotany and Pharmacognosy Lab, Department of Botany, University of Chittagong, Chittagong 4331, Bangladesh; helaluddinchowdhurycu@gmail.com; 4 Department of Microbiology, University of Chittagong, Chittagong 4331, Bangladesh; tuhin.mbio@gmail.com; 5 Department of Pharmacy, BGC Trust University Bangladesh, Chittagong 4381, Bangladesh; arka.bgctub@gmail.com (A.P.); talhabmb@bgctub.ac.bd (T.B.E.); 6 Department of Nutrition and Dietetics, Faculty of Health Sciences, Gazi University, Emek, Ankara 06490, Turkey; duyguturkozu@gazi.edu.tr; 7 Department of Agricultural Sciences, University of Naples Federico II, 80055 Portici, Italy; 8 Nutrition and Bromatology Group, Department of Analytical and Food Chemistry, Faculty of Food Science and Technology, University of Vigo—Ourense Campus, E32004 Ourense, Spain

**Keywords:** *Molineria capitulata*, medicinal plants, bioactive molecules, biological activity, antioxidant, thrombolytic, anti-inflammatory, computational study

## Abstract

*Molineria capitulata* is an ornamental plant that has traditionally been used to treat several chronic diseases. The present study was designed to examine the antioxidant, cytotoxic, thrombolytic, anti-inflammatory, and analgesic activities of a methanolic extract of *M. capitulata* leaves (MEMC) using both experimental and computational models. Previously established protocols were used to perform qualitative and quantitative phytochemical screening in MEMC. A computational study, including molecular docking and ADME/T analyses, was performed. The quantitative phytochemical analysis revealed the total phenolic and flavonoid contents as 148.67 and 24 mg/g, respectively. Antioxidant activity was assessed by examining the reducing power of MEMC, resulting in absorbance of 1.87 at 400 µg/mL, demonstrating a strong reduction capacity. The extract exhibited significant protection against blood clotting and showed the highest protein denaturation inhibition at 500 µg/mL. In both the acetic acid-induced writhing and formalin-induced paw-licking models, MEMC resulted in significant potential pain inhibition in mice. In the computational analysis, 4-hydroxybenzaldehyde, orcinol glucoside, curcapital, crassifogenin C, and 2,6-dimethoxy-benzoic acid displayed a strong predictive binding affinity against the respective receptors. These findings indicated that *M. capitulata* possesses significant pharmacological activities to an extent supported by computational studies.

## 1. Introduction

Treatments with synthetic drugs are often associated with higher side effects and cannot be tolerated by some people at high doses. Conversely, traditionally important medicinal plant species have been used to treat a variety of diseases, although their underlying mechanisms remain unknown. Since ancient times, plant extracts have been used to treat various infections, and herbal treatments remain popular due to lower costs and less harmful characteristics compared with synthetic drugs [1].

Free radical-mediated cell damage has been associated with a variety of disorders, including cancer, arthritis, and diabetes. Some free radicals and reactive nitrogen species can trigger and upregulate cell death mechanisms, including apoptosis, and free radicals have been associated with the excessive occurrence of necrosis. Antioxidants have been associated with a variety of medicinal attributes; for example, the mitochondria-targeted ubiquinone may reduce alcohol-induced liver damage. Several experts have proposed that antioxidants could alleviate free radical production and could enhance efficacy of drugs [2].

Thrombosis can be defined as a process of a blood clot that forms within the blood vessel and can block the blood flow in the affected area and ultimately be responsible for the formation of various diseases such as pulmonary emboli, deep vein thrombosis, strokes, and heart attacks. Alternatively, thrombolytic agents are those agents that prevent the formation of a blood clot in blood vessels, such as tissue plasminogen activator (tPA), streptokinase (SK), urokinase, etc. In this case, natural thrombolytic agents that come from traditionally important medicinal plants could be a good source, as those agents have fewer side effects compared to synthetic drugs. Nowadays, more than 60% of cytotoxic agents have been obtained from natural sources including plants, marine organisms, and microorganisms, either directly or by chemical alteration that relied on natural lead compounds. Moreover, natural products have a wide range of use in cancer chemotherapy.

Unexpected conditions and disease can often cause direct (physical) or indirect (mental) distress associated with pain. Pain is processed by the central nervous system by specialized neurons called nociceptors, which send pain-related information to the brain in response to noxious stimuli [3,4]. Acute or severe pain can be induced by the synthesis of prostaglandins, serotonin, and cyclooxygenase COX-I and II. On the other hand, inflammation is a common underlying cause of injury, inducing a complicated network of enzyme activations that can mediate fluid extravasation, cell migration, cell disruption, and restoration. NSAIDs are frequently prescribed medicines because they can be widely applied to the treatment of pain, inflammation, fever, and rheumatic diseases. However, NSAIDs can cause adverse effects in the digestive tract, including dyspeptic symptoms, gastrointestinal erosions, and peptic ulcers that can lead to perforation and bleeding. To reduce the toxicity of these drugs, the identification and development of novel anti-inflammatory medicines are essential, and natural products, such as those derived from medicinal plants, represent a potential source of new drugs [3,5].

Worldwide, the medicinal features of several plants have been sought due to their effective pharmacological effects. The genus *Molineria* contains at least seven identified species, all of which have been utilized for a variety of medicinal purposes across multiple cultures for many years. The *M. capitulata* rhizome is often employed in Chinese traditional medicine for the treatment of consumptive cough, spermatorrhea, impotence, and kidney asthenia. In India, this plant has several traditional uses, including the treatment of jaundice, gonorrhea, colic, asthma, diarrhea, urinary tract infection, cystitis, rheumatic arthritis, acute renal pelvis, nephritis, nephritis-edema, and hypertension. *M. capitulata* is a small herb and is recognized as an ornamental flowering plant that belongs to the Hypoxidaceae family [6].

Although *M. capitulata* has a variety of traditional uses globally, until now no scientifically detailed reports exist regarding its pharmacological activities. Based on its long history of traditional use, we examined *M. capitulata* to assess its antioxidant, cytotoxic, thrombolytic, anti-inflammatory, and analgesic potential and to identify the potential phytochemical constituents responsible for those effects using computational modeling.

## 2. Materials and Methods

### 2.1. Plant Material

From hills of the Sitakunda area of the Chittagong district, Bangladesh, newish, suppurated *M. capitulata* leaves were collected and identified by Shaikh Bokhtear Uddin, Taxonomist and Professor in the Department of Botany, University of Chittagong, Chittagong-4331, Bangladesh.

### 2.2. Preparation of Extracts

Collected plant leaves were maintained in a dry, clean environment, with access to natural sunlight. When the leaves were dried completely, they were ground into a powdered form. After completing the measurement, 250 g of the ground leaf powder was soaked in methanol (2 L) at room temperature for 7 days with occasional stirring and shaking. The extract was then filtered, first through cotton wodge and then through Whatman no. 1 filter paper. The residual solvent was subjected to evaporation with a water bath at 60 °C temperature to yield the crude methanol extract, and the plant crude extract was obtained as a semisolid (30.55 g dry weight, yield: 5.09% *w/w*). The obtained methanolic extract (MEMC) was used in the present experiments, and all other residues were stored at 4 °C for later use.

### 2.3. Chemicals

Lyophilized streptokinase (1,500,000 IU) was obtained from Square Pharmaceuticals Ltd., Dhaka, Bangladesh. Gallic acid, quercetin, and ferric chloride were obtained from Sigma Chemical Co., Saint Louis, MI, USA. Potassium ferricyanide, sodium carbonate, and ascorbic acid were purchased from Merck KGaA, Frankfurt, Darmstadt, Germany, and SD Fine Chem. Ltd., Kolkata, West Bengal, India. Ascorbic acid was purchased from SD Fine Chem. Ltd., Kolkata, West Bengal, India, and potassium ferricyanide from May and Backer, Dagenham, UK. Methanol, Folin-Ciocalteu reagent, sodium carbonate, and potassium ferricyanide were purchased from Merck KGaA, Frankfurt, Darmstadt, Germany. All chemicals were of analytical reagent grade.

### 2.4. Qualitative Phytochemical Screening of MEMC

Primary phytochemical analysis of MEMC was performed using standard chemicals: Mayer’s and Wagner’s reagents were used to determine alkaloid contents; Molisch reagent was used to determine carbohydrate contents; ferric chloride and lead acetate were used to determine phenol and tannin contents, respectively; Fehling’s and Benedict’s solution were used to determine the reducing power; and a frothing test was performed using saponin. All tests were performed using standard protocols [7]. The color intensity or precipitate formation were assessed as the analytical responses to qualitative tests. Each test was performed using a 10% (*w/v*) methanol extract solution.

### 2.5. Quantitative Phytochemical Screening of MEMC

#### Total Phenolic Content (TPC) Determination

The total phenolic content (TPC) in MEMC was ascertained using the Folin–Ciocalteu reagent (FCR) [7]. A volume of 1 mL extract solution was combined with 2.5 mL FCR and 2.5 mL Na_2_CO_3_ and incubated at 25 °C for 20 min. The absorbance was measured at 760 nm using an ultraviolet (UV) spectrophotometer. Several known concentrations of gallic acid were used to generate a standard curve, which was used to derive an equation for ascertaining the TPC (expressed as mg gallic acid equivalents (GAE)/g per extract), as follows (Equation (1)).

y = 0.0039x + 0.033, R^2^ = 0.9979 (1)

where x is the absorbance and y is the gallic acid equivalent (in mg/g).

### 2.6. Total Flavonoid Content (TFC) Determination

The total flavonoid contents (TFC) of MEMC were ascertained using a previously described protocol [8]. A 1 mL volume of extract solution was combined with 1 mL 2% AlCl_3_ in methanol and incubated for 15 min. The absorbance was measured spectrophotometrically at 430 nm. Quercetin was used to build a standard curve, and TFC was calculated (as µg Quercetin equivalent/mg dry material) using the following equation (Equation (2)).

y = 0.034x + 0.0475, R^2^ = 0.9979 (2)

where x is the absorbance and y is the quercetin equivalent (in µg/mg).

### 2.7. In Vitro Antioxidant Effect

#### Ferric Reducing Antioxidant Power Assay

The ferric reducing antioxidant power assay was performed according to a previously established protocol [9]. Various concentrations of MEMC (0.25–1 mg/mL) were adjusted to a 2.5 mL volume and combined with 2.5 mL 0.2 M phosphate buffer (pH 6.6), and 2.5 mL 1% aqueous potassium hexacyanoferrate [K_3_Fe(CN)_6_] solution. After 20 min of incubation at 50 °C, 2.5 mL of 10% trichloroacetic acid (TCA) was added to the mixture, and the mixture was centrifuged at 3000 rpm for 10 min. The upper phase (2.5 mL) was extracted and mixed with distilled water (2.5 mL) and 0.5 mL 0.1% FeCl_3_. The absorbance was determined spectrophotometrically at 700 nm. The reducing power was reported as ascorbic acid equivalents per mg extract. A blank solution was used without the addition of sample.

### 2.8. Brine Shrimp Cytotoxicity

Brine shrimp lethality was ascertained using a previously established protocol [10]. Hatched brine shrimp and saline (prepared using sea salt, a small amount of dimethyl sulfoxide (DMSO), and distilled water) were used for this test. Extract dilutions ranging from 31.25 to 1000 µg/mL were generated from a 5 mg extract solution using a small amount of DMSO and placed in test tubes. Then, brine shrimp were added to each tube. The amount of shrimp death was determined after 24 h, and the percent mortality was calculated using Equation (3), as follows. Here, vincristine sulfate was used as a reference standard.

% of mortality = (N_0_ − N_1/_N_0_) × 100 (3)

where N_0_ = the starting number of nauplii; N_1_ = the number of dead nauplii.

### 2.9. Thrombolytic Activity

Thrombolytic activity was ascertained using a previously established protocol [11,12]. A stock solution of lyophilized streptokinase (1,500,000 IU) was dissolved in 5 mL sterile distilled water. Blood was obtained from healthy individuals (*n* = 10) using a syringe, and 0.5 mL aliquots were prepared in pre-weighed Eppendorf tubes, followed by incubation for 45 min at 37 °C. Serum produced in the upper level of the tube was carefully removed using a syringe without disturbing the clot. Then, measurements were performed to ascertain the degree of clotting. Plant extract solution (10 mg/mL), 100 µL positive control (Streptokinase), and 100 µL negative control (saline) were added to separate blood-containing Eppendorf tubes and incubated for up to 90 min at 37 °C. The produced serum was removed, and the tube was measured again to differentiate the weight without disrupting the clot. The percent clot lysis activity was measured by the following equation (Equation (4)).

% of clot lysis = (weight of clot after fluid removal/clot weight) × 100(4)

### 2.10. In Vitro Anti-Inflammatory Activity

Anti-inflammatory MEMC activity was ascertained using a previously established albumin denaturation protocol, with slight modifications [13,14]. The preparation of the test solution (0.5 mL) included the test sample (0.05 mL) and 0.45 mL 5% (*w/v*) aqueous albumin solution prepared in distilled water (0.5 mL). MEMC and the standard drug (diclofenac sodium) were tested at 250 and 500 µg/mL within individual test tubes, and the pH was adjusted to 6.3 with 1 N HCl. The samples were incubated at 37 °C for 20 min, followed by 3 min at 57 °C. After the samples returned to room temperature, the absorbance was measured by a UV spectrophotometer at 416 nm. The negative control included all chemicals without the addition of the extract or drug standard. The percentage of protein denaturation was quantified using the following equation (Equation (5)).

% of protein denaturation = [(Ac − As)/Ac] × 100(5)

where Ac = absorbance of control and As = absorbance of sample.

### 2.11. In Vivo Analgesic Activity

#### 2.11.1. Experimental Animals and Ethical Statement

Swiss Albino mice weighing 25–30 g were obtained from the International Center for Diarrheal Diseases Research, Bangladesh (ICDDR, B) and warehoused in polypropylene crates under specific environments. The animals were housed under conditions of a 12-h: 12-h light:dark photoperiod, with an ambient temperature of 26 ± 2 °C, with *ad libitum* access to drinking water and a food pellet diet. Mice were allowed to adapt for seven days prior to experiments. The experimental animal protocols used in this study were approved by the Institutional Animal Ethics Committee, Department of Pharmacy, International Islamic University Chittagong, Chittagong 4318, Bangladesh (Ref. No.: Pharm-P&D-80/06′19-152).

#### 2.11.2. Acetic Acid-Induced Writhing Method

The acetic acid-induced writhing protocol was performed as previously described [15], with some modifications. For pain-inducing purposes, acetic acid was administered intraperitoneally (IP) in experimental animals. Mice were randomly divided into four groups, containing six mice per group. Group I received distilled water, Group II received diclofenac sodium at 10 mg/kg, and Groups III and IV were treated with MEMC at 200 and 400 mg/kg, respectively, after an overnight fast. Test samples were administered orally 30 min after the intraperitoneal administration of 0.7% (*v/v*) solution of acetic acid. All mice were housed individually to allow for proper observation. Each mouse was independently observed to obtain a writhing calculation for the 10-min period starting 5 min after the IP administration of acetic acid. Sometimes full writhing was not observed, or the mice presented writhing behavior but not fully. These were counted as half writhing behaviors, and two observations of half writhing behavior were counted as one full writhing. The numbers of writhing behaviors counted in each group were compared against that of the control group, using diclofenac sodium as the standard drug treatment (positive control). The percentage of writhing inhibition was calculated using Equation (6).

Percentage of inhibition (%) = [(VC − VT)/VC] × 100(6)

where VT = number of writhing motions in extract-treated mice and VC = number of writhing motions in the control group.

#### 2.11.3. Formalin-Induced Writhing Test

Mice were fed with the extract solution at 200 or 400 mg/kg body weight. After 30 min, 20 µL 2.5% formalin solution was administered to a subcutaneous area of the right paw. Pain sensation is manifested in mice by biting or licking behavior. Mouse behaviors were captured on video for 30 min after formalin administration. The 0–5-min period was considered the neurogenic or early-phase period, whereas the inflammatory or late-phase period was considered to be represented by the 15–30-min time point. The percentage of inhibition was ascertained as described for the acetic acid protocol [16].

### 2.12. In Silico Analysis

#### 2.12.1. Selection of Compounds for the Computational Study

We identified 39 compounds in *M. capitulata* by performing a literature review [6]. The inclusion criteria were: research and review articles published associated with *M. capitulata* [6]. Articles that did not match the above criteria were excluded from the study. Later, we performed an ADME study for all 39 compounds and found that only ten compounds satisfied the criteria as given by the Lipinski’s rule of five. According to Lipinski’s rule, we have selected only 10 compounds for further computational study, namely in silico molecular docking analysis, where the name of our selected compounds were as follows: 3-(4-hydroxy-3-methoxyphenyl) acrylaldehyde (PubChem ID: 5280536), 4-hydroxybenzaldehyde (PubChem ID: 126), orcinol glucoside (PubChem ID: 12315192), pilosidine (PubChem ID: 10096608), capituloside (PubChem ID: 3013844), curcapital (PubChem ID: 10733722), crassifogenin C (PubChem ID: 70680261), breviscaside A (PubChem ID: 101497745), methyl-4-O-coumaroylquinate (PubChem ID: 11631689), and 2,6-dimethoxy-benzoic acid (PubChem ID: 15109).

#### 2.12.2. ADME/T Analysis

The pharmacokinetic characteristics of each compound were easily ascertained by the SwissADME online server [17]. In this study, several molecular descriptors were obtained, including molecular weight, molar refractivity, lipophilicity, hydrogen bond donors, hydrogen bond acceptors, numbers of rotatable bonds, percentage of absorption, topological polar surface area, and violations of Lipinski’s rule of five. Active drugs that are administered orally must also comply with extensively exploited drug-likeness features to determine the compounds’ pharmaceutical reliability. The toxicological characteristics of each compound are also important factors, which were determined by using the admetSAR online server [17], as described previously. Because toxicity is a major issue for drug discovery, we assessed Ames toxicity, carcinogenic properties, acute oral toxicity, and acute rat toxicity in our study.

#### 2.12.3. Molecular Docking Analysis

Molecular docking was performed using Glide from Schrödinger Maestro (version 10.1, Schrödinger, LLC New York, NY, USA) to predict the active potential of compounds of MEMC against the active sites of receptors (1HD2, 1AH5, 2OYE, 6COX), which were identified through a literature review.

#### 2.12.4. Ligand and Protein Preparation

Based on the literature review, we chose 10 major compounds found in MEMC and obtained all structure-related information for these identified compounds from the PubChem database [17]. Three dimensional (3D) crystallographic structures of all proteins were obtained from the Protein Data Bank RCSB PDB: human peroxiredoxin 5 (PDB ID: 1HD2), a novel type of mammalian peroxiredoxin for antioxidant activity; tissue-type plasminogen activator (PDB ID: 1A5H) for thrombolytic activity; cyclooxygenase-1 in complexed with Indomethacin-(R)-alpha-ethyl-ethanolamide (PDB ID: 2OYE); and cyclooxygenase-2 in complexed with a selective inhibitor, sc-558 in i222 space group (PDB ID: 6COX) for analgesic and anti-inflammatory activities. Each compound was structurally imprinted in three dimensions (3D) by applying Ligprep 2.5 in the Schrödinger Suite, 2013, and the ionization state was originated at pH 7.0 ± 2.0 by applying Epik 2.2 in the Schrödinger Suite. To prepare the protein preparation structures for molecular docking analysis, the 3D images of each protein were obtained from the Protein Data Bank. All structures were propagated and filtered by the protein preparation wizard in Schrödinger Maestro (version 10.1). All charges and bond orders were attributed, hydrogen was added to heavy atoms, selenomethionine was replaced by methionine, water was eliminated, and a force field was maintained in OPLS_2005.

#### 2.12.5. Receptor Grid Generation

Grid generation retained the default factors of van der Waals scaling parameters at 1.00 and charge break-off inhibition at 0.25 to maintain the force field in OPLS_2005. A bounding box (15 Å × 15 Å × 15 Å) of unmarked dimensions was centered within the centroid active site/ligand activation site that was produced for the enzyme/receptor. The identified active binding site remained on the protein target site.

#### 2.12.6. Glide Standard Precision Ligand Docking

The docking study was conveyed by Schrödinger Maestro (version 10.1), between which amercement was used to non-cis/trans amide bonds. Glide docking was accomplished using Van der Waals scaling parameters and half of charge. A gradual intercept from 0.80 to 0.15 was chosen for the ligand atoms. Conclusive scoring was customized for energy-minimized poses and presented as docking scores. The minimum glide score was determined according to the best-docked pose, and the value was recorded for each ligand.

### 2.13. Statistical Analysis

The results are presented as the mean ± SEM (standard error mean) of three animals. Statistical analyses were performed using the Statistical Package for the Social Sciences (SPSS) and a one-way analysis of variance (ANOVA), followed by a post hoc Dunnett’s (*t*-test), which was used for comparisons between the test samples and the control (Tween-80). A *p*-value < 0.001 compared with the control was considered significant. All diagrams were generated using Graph Pad Prism version 8.0.

## 3. Results

### 3.1. Qualitative Phytochemical Screening

Various phytochemical secondary metabolites in MEMC were identified, including alkaloids, proteins, flavonoids, saponins, and phenolic compounds, and the results can be found in Table 1.

### 3.2. Quantitative Phytochemical Screening

#### Determination of Total Phenolic Content (TPC) and Total Flavonoid Content (TFC)

The TPC in MEMC is expressed in GAE and was determined to be 148.67 ± 1.15 mg GAE/g of dry extract (Table 2). The TFC is expressed in QE and was determined to be 24.00 ± 0.00 mg QE/g dry extract (Table 2).

### 3.3. Evaluation of In Vitro Antioxidant Effect

#### Reducing Power Capacity

**Figure 1:**
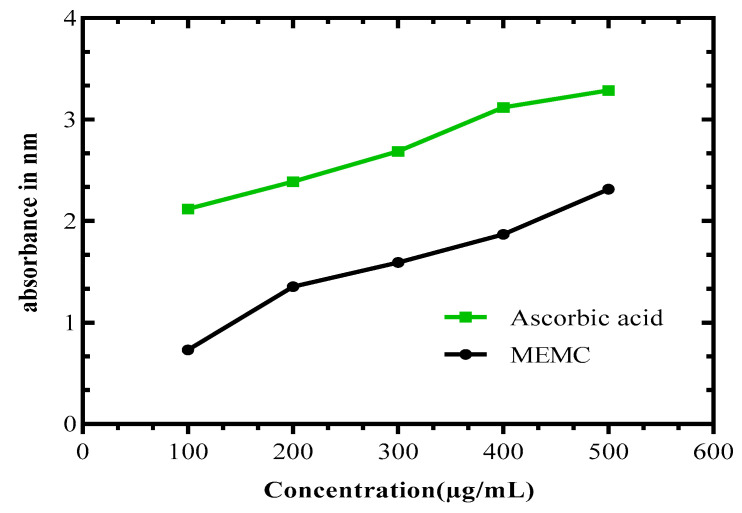
Reducing power capacity of MEMC compared with the reference standard ascorbic acid. MEMC, methanol leaf extract of *Molineria capitulata.*

MEMC had noteworthy reducing power capacity. Here, the absorbance is directly proportional to the concentrations of the MEMC. The extract amplified the absorbance significantly, indicating the antioxidant potential of MEMC, with absorbance measured at 1.871 and 0.732 for the 400 and 100 µg/mL concentrations, respectively. The reducing power was analogous to the activity of the reference standard, ascorbic acid, which resulted in absorbance values of 3.12 and 2.12 at identical concentrations (Figure 1).

### 3.4. Brine Shrimp Cytotoxicity

**Figure 2:**
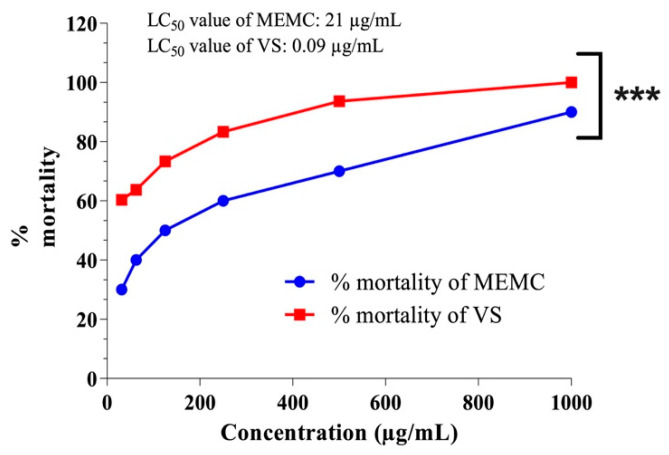
Determination of LC50 value of MEMC and VS (vincristine sulfate) from linear correlation between concentrations versus percentage of mortality. Values are expressed as mean ± SEM (*n* = 3) where data were analyzed by two-way ANOVA (Sidak’s multiple comparison test) and *** *p* value less than 0.001 is considered as statistically significant. MEMC, methanol leaf extract of Molineria capitulata.

The results of the cytotoxicity assay are reported as the percentage of nauplii death recorded at different MEMC concentrations. According to this assay, MCME demonstrated a 50% lethal concentration (LC_50_) value of 21 µg/mL, which was significantly different from the value for the positive control vincristine sulfate, which has an LC_50_ value of 0.09 µg/mL, indicating the moderate toxicity of the analyzed extract (Figure 2).

### 3.5. Thrombolytic Activity

**Figure 3:**
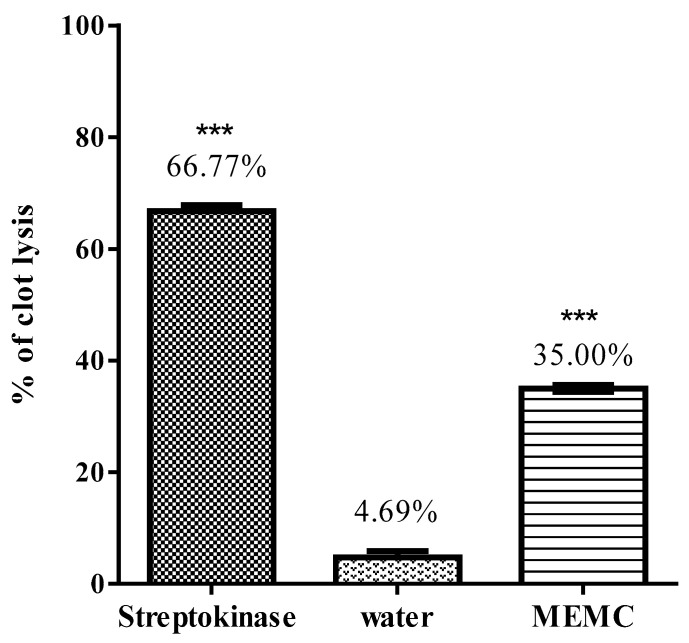
Thrombolytic activity of MEMC (methanol leaf extract of Molineria capitulata). Percentage of clot lysis of MEMC in comparison with negative control (water). Values are expressed as mean ± SEM for 10 volunteers, where data were analyzed by Dunnett’s test and *** *p* value less than 0.001 is considered as statistically significant.

Streptokinase produce a significant (*p* < 0.0001) percentage of clot lysis (66.77 ± 1.08%) compared with water (4.69 ± 1.19%). After the treatment of clots with 100 µL MEMC, a significant level (36.818 ± 0.58%) of clot lysis was observed (Figure 3).

### 3.6. Anti-Inflammatory Activity

#### Inhibition of Protein Denaturation

**Figure 4:**
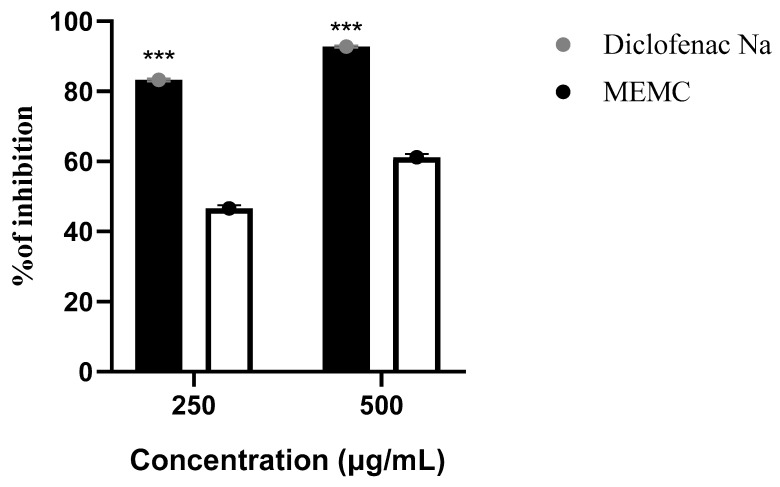
Anti-inflammatory activity of MEMC (methanol leaf extract of Molineria capitulata). Percentage of inhibition of protein denaturation of MEMC in comparison with reference standard (Diclofenac Na). Values are expressed as mean ± SEM (*n* = 3) where data were analyzed by Sidak’s multiple comparison test and *** *p* value less than 0.001 is considered as statistically significant.

The anti-inflammatory activity of MEMC was evaluated using the protein denaturation method, which is summarized in Figure 4. The results demonstrated the significant inhibition of protein denaturation by MEMC compared with the positive control, diclofenac sodium. The anti-inflammatory activities showed a dose-dependent response, with MEMC treatment resulting in the inhibition of 61.22 ± 0.87% and 46.66 ± 0.88% of protein denaturation at 500 and 250 μg/mL, respectively. In contrast, the positive control diclofenac sodium showed a maximal level of 92.77 ± 0.34% inhibition at a dose of 500 μg/mL.

### 3.7. In Vivo Analgesic Effect

#### 3.7.1. Acetic Acid-Induced Writhing Test

The oral administration of both doses of MEMC significantly attenuated the acetic acid-induced abdominal writhing of mice in a dose-dependent fashion (Table 3). The percent inhibition of the writhing response of MEMC and the standard drug diclofenac sodium (10 mg/kg) were compared against the negative control.

#### 3.7.2. Formalin-Induced Writhing Test

Both 200 and 400 mg/kg concentrations of MEMC significantly inhibited the licking responses during the early and late phases. In the early phase, the percentages of inhibition were 21.78% and 51.67%, whereas the standard drug (10 mg/kg) resulted in a percentage of inhibition of 75.69% relative to the negative control. During the late phase, the percentages of inhibition for MEMC were 24.32% and 53.60%, whereas the value for the standard drug (10 mg/kg) was 62.16% relative to the negative control (Table 4).

### 3.8. In Silico Analysis

#### 3.8.1. Pharmacokinetic Property and Toxicological Properties Analysis

The compliance of our selected compounds with Lipinski’s rule of five was evaluated according to the pharmacokinetic characteristics and was evaluated by an online site. The ADME (absorption, distribution, metabolism, elimination) characteristics of the chosen compounds are displayed in Table 5. All identified characteristics were associated with cell permeation, bioavailability, and metabolism. The compounds that displayed properties within acceptable limits were viewed as having good bioavailability. The toxicological properties of the selected compounds were also predicted using the admetSAR online tool, and the results are shown in Table 6. All of the evaluated compounds showed non-Ames toxicity and non-carcinogenic properties.

#### 3.8.2. Molecular Docking Analysis Associated with Antioxidant Activity

**Figure 5:**
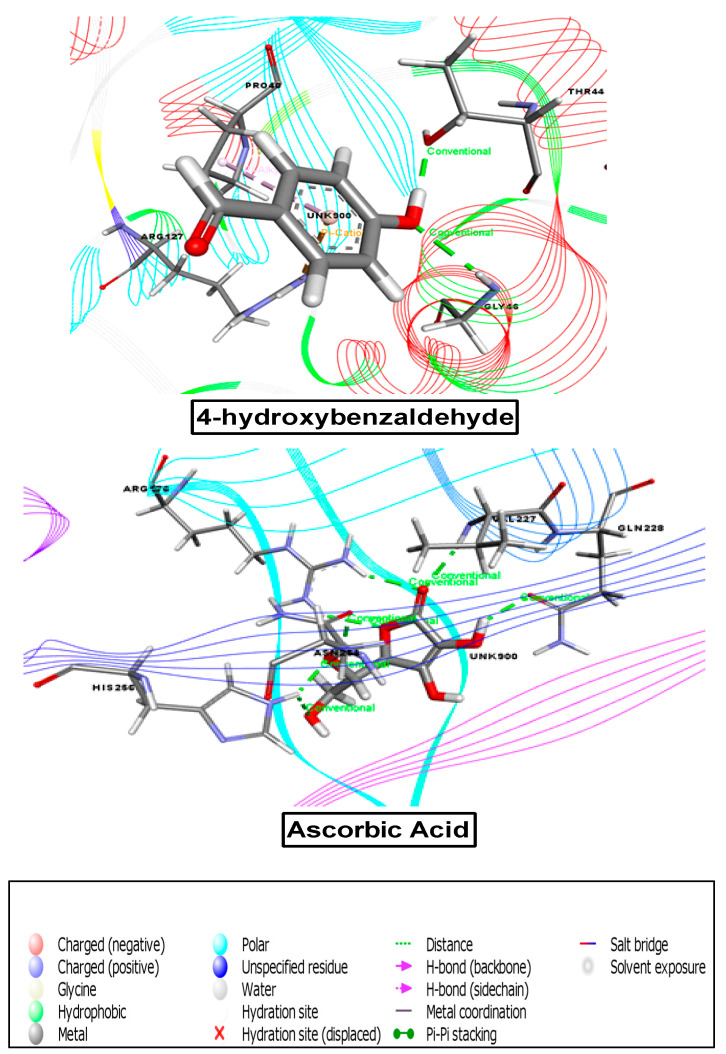
Best ranked pose of the best compound of *M. capitulata*. 4-hydroxybenzaldehyde and standard drug (ascorbic acid) in the binding pocket of human peroxiredoxin 5 (PDB ID: 1HD2) for antioxidant activity.

To determine the probable antioxidant effects, we used the 1HD2 receptor in a molecular docking analysis against all ten compounds, among which nine compounds exhibited great binding affinity. According to the docking score, the binding affinities from maximum to minimum were as follows: breviscaside A (−2.699), crassifogenin C (−2.863), pilosidine (−3.272), capituloside (−3.272), orcinol glucoside (−3.543), 2,6-dimethoxy-benzoic acid (−3.686), curcapital (−4.259), methyl-4-O-coumaroylquinate (−4.599), and 4-hydroxybenzaldehyde (−5.253) (Table 7 and Figure 5).

#### 3.8.3. Molecular Docking Analysis Associated with Thrombolytic Activity

**Figure 6:**
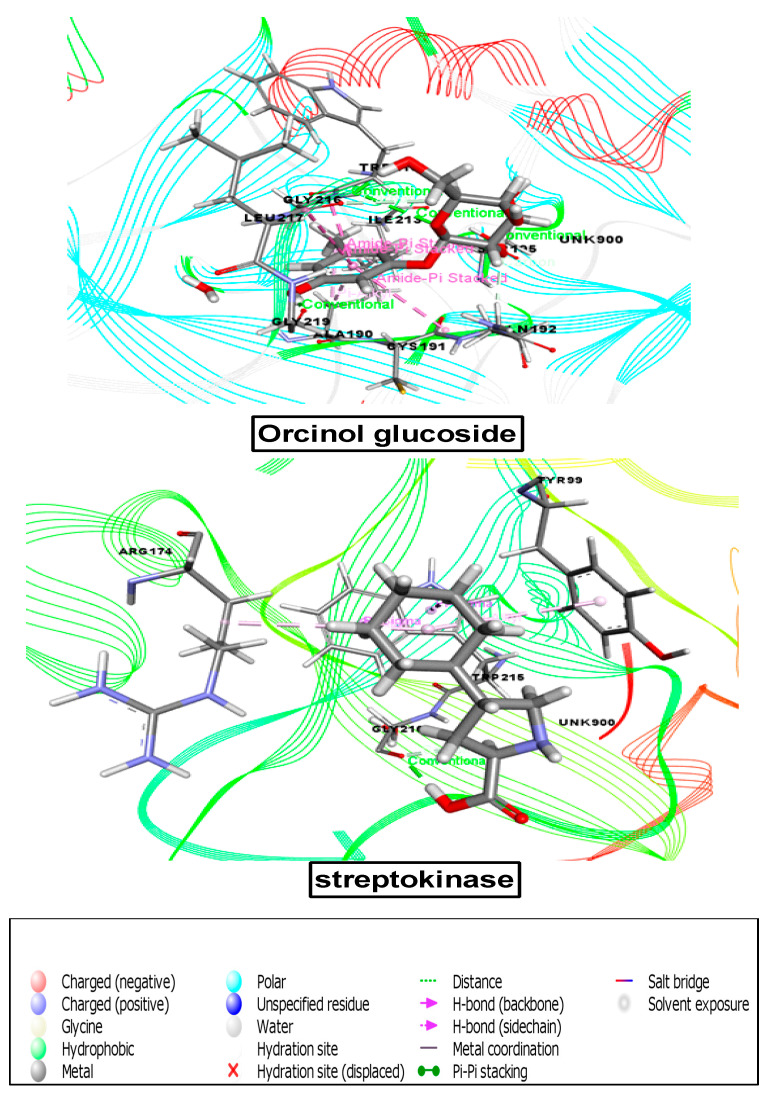
Best ranked pose of the major compounds of *M. capitulata*. Orcinol glucoside and standard drug (streptokinase) in the binding pocket of human tissue-type plasminogen activator (PDB ID: 1A5H) for thrombolytic activity.

In the thrombolytic study, a known receptor, 1A5H, was compared against all compounds to determine probable activity based on the docking score. According to the docking score, the binding affinities from maximum to minimum were as follows: 3-(4-hydroxy-3-methoxyphenyl)acrylaldehyde (−2.02), pilosidine (−3.957), capituloside (−3.957), breviscaside A (−4.29), crassifogenin C (−4.612), methyl-4-O-coumaroylquinate (−4.75), 2,6-dimethoxy-benzoic acid (−5.618), curcapital (−5.857), 4-hydroxybenzaldehyde (−6.00), and orcinol glucoside (−6.547) (Table 7 and Figure 6).

#### 3.8.4. Molecular Docking Analysis Associated with Analgesic Activity

**Figure 7:**
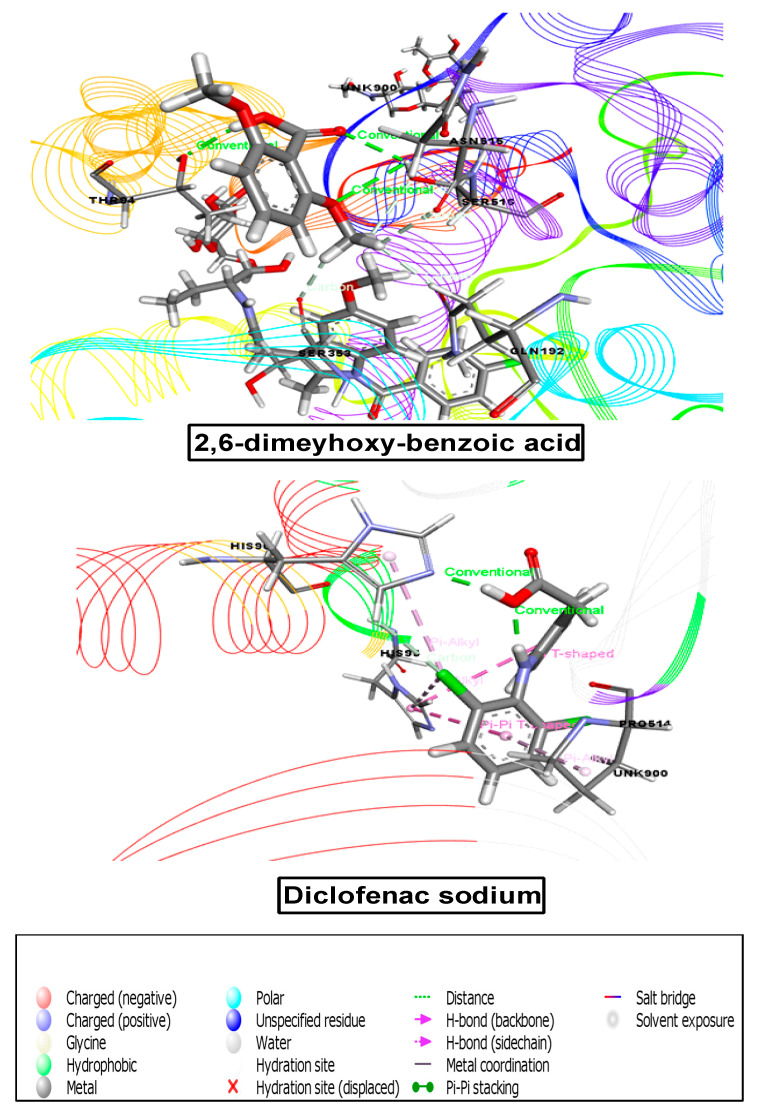
Best ranked pose of the major compounds of *M. capitulata*. 2,6-dimeyhoxy-benzoic acid and standard drug (diclofenac sodium) in the binding pocket of COX-1 enzyme (PDB ID: 2OYE) for analgesic and anti-inflammatory activities.

For the determination of analgesic effects, the receptor 2OYE was docked against these ten compounds, all of which demonstrated good binding scores. According to the docking score, the binding affinities from maximum to minimum were as follows: methyl-4-O-coumaroylquinate (−3.875), orcinol glucoside (−4.163), pilosidine, (−4.436), capituloside (−4.69), crassifogenin C (−4.859), 3-(4-hydroxy-3-methoxyphenyl)acrylaldehyde (−5.194), breviscaside A (−5.255), curcapital (−5.321), 4-hydroxybenzaldehyde (−5.413), and 2,6-dimethoxy-benzoic acid (−5.922) (Table 7 and Figure 7).

#### 3.8.5. Molecular Docking Analysis Associated with Anti-Inflammatory Activity

**Figure 8:**
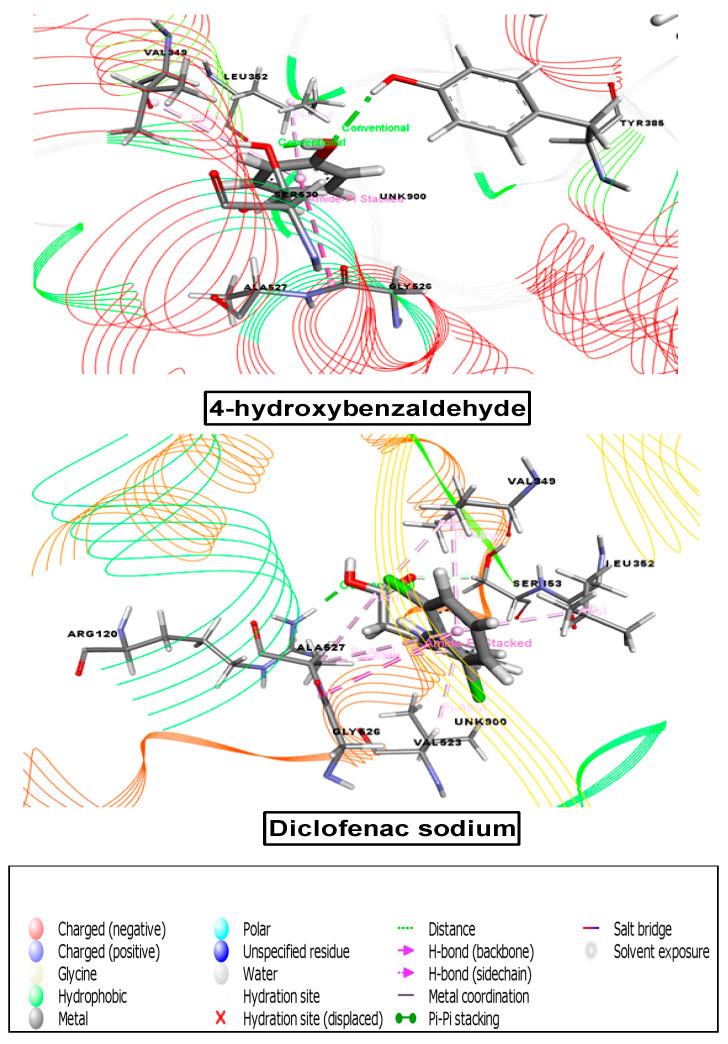
Best ranked pose of the major compounds of *M. capitulata.* 4-hydroxybenzaldehyde and standard drug (diclofenac sodium) in the binding pocket of COX-2 enzyme (PDB ID: 6COX) for analgesic and anti-inflammatory activities.

**Figure 9:**
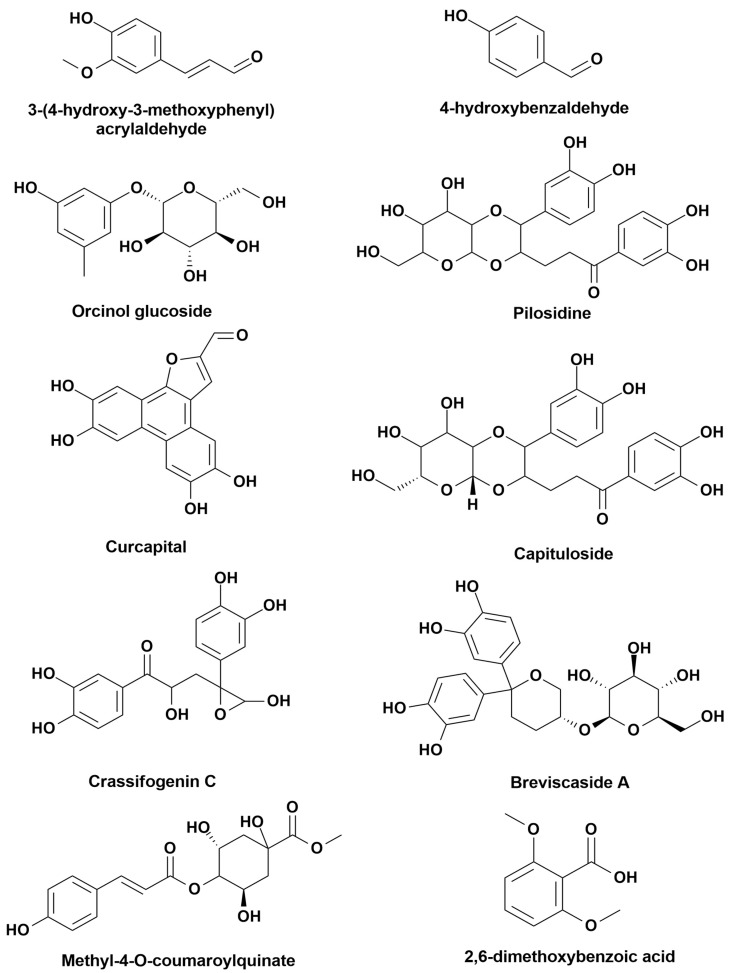
Chemical structures of the compounds used for molecular docking study.

For the anti-inflammatory study, our chosen receptor, 6COX, was docked against all ten compounds, and seven compounds demonstrated great binding affinity. According to the docking score, the binding affinities from maximum to minimum were as follows: orcinol glucoside (−5.266), methyl-4-O-coumaroylquinate (−5.913), 3-(4-hydroxy-3-methoxyphenyl)acrylaldehyde (−6.26), 2,6-dimethoxy-benzoic acid (−7.11), 4-hydroxybenzaldehyde (−7.464), crassifogenin C (−8.204), and curcapital (−9.323) (Table 7, Figure 8 and Figure 9).

## 4. Discussion

Our study was performed to ascertain the analgesic, antioxidant, cytotoxic, thrombolytic, and anti-inflammatory activities of MEMC using experimental (in vivo and in vitro assays) and computational (molecular docking and ADME/T analysis) approaches. The qualitative phytochemical screening revealed that MEMC contains several phytoconstituents, including alkaloids, carbohydrates, flavonoids, phenols, tannins, saponins, reducing sugars, glycosides, and terpenoids [18,19,20,21]. Our quantitative phytochemical assay illustrated that MEMC has a TPC of 148.67 mg GAE/g and a TFC of 24 mg QE/g. Our plant extract contains a variety of phytoconstituents, suggesting the potential to display numerous pharmacological properties, including analgesic, antioxidant, cytotoxic, thrombolytic, and anti-inflammatory activities, among others. Analgesic, antioxidant, and anti-inflammatory activities have been directly associated with phenolic compounds [22,23]. Phenolic compounds have also been shown to demonstrate analgesic, antibacterial, antioxidant, anticancer, anti-inflammatory, and antimicrobial properties. Acute oral toxicity assessments of MEMC did not result in any unusual behaviors, fatalities, or neurological disturbance until the administration of a 2000 mg/kg dose, indicating that MEMC presents relatively low toxicity and can be considered safe at doses of up to 2000 mg/kg.

Antioxidants reduce free radicals, preventing free radical-induced damage by donating electrons to neutralize free radical formation, improving stability. Some foods (fruits, vegetables, herbs, and cereals) and plant extracts have been found to contain large quantities of phenolics, which have received increasing attention from the food industry due to their antioxidant features, which can also increase the nutritional value of foods. Recent understanding of free radical mechanisms has had a large impact on the dietary sector because antioxidants have been found to have beneficial effects in several human illnesses, including cancer, stroke, atherosclerosis, arthritis, diabetes, and neurodegenerative diseases. Antioxidants can inhibit or slow the oxidation of lipids and other molecules by converting the extension of the oxidation chain mechanism. The reducing capacity is associated with the presence of a reductant, which can apply antioxidant mechanisms to convert Fe^3+^ (ferricyanide) to Fe^2+^ (ferrous) [24], allowing for the antioxidant potentiality of plant polyphenols to easily be measured by performing the reducing power test. Our plant extract was measured at an absorbance of 2.313 compared with the ascorbic acid (standard) value of 3.287 at the same concentration (500 µg/mL). Several phenolic components, including flavonoids, phenolic diterpenes, and phenolic acids, have antioxidant properties. Our plant extract contained large quantities of phenols and flavonoids, which indicated that *M. capitulata* has a high reducing capacity and the ability to disrupt free radical chains.

Medicinal plants are reliable sources of new chemotherapeutic agents, and the brine shrimp lethality assay is one method that can be used to determine the lethality of a compound [25]. In this assay, vincristine sulfate was used as a reference standard. Vincristine is an anti-cancer (antineoplastic/cytotoxic) chemotherapy drug that belongs to a group of drugs called ‘’vinca’’ alkaloids. Vincristine works by stopping the cancer cells from separating into two new cells. Thus, it stops the growth of the cancer. This test is considered to be a fast, easy, and accessible bioassay technique for evaluating anticancer and cytotoxicity properties and can be used as a reference for pesticides and compounds with antiviral, antibacterial, antimalarial, and antitumor properties. During the brine shrimp lethality assay, cytotoxicity is ascertained as impotent when the LC_50_ value is in the range of 500–1000 µg/mL, average at 100–500 µg/mL, and potent at 0–100 µg/mL. In our study, several concentrations (31.25–1000 µg/mL) of MEMC and vincristine sulfate were examined to ascertain their cytotoxic effects, as presented in Figure 2. We identified a moderately toxic effect for our plant extract compared with that of the standard drug (vincristine sulfate). These toxicity effects might due to the secondary metabolites produced by bioactive compounds in our plant extract, some of which have previously been associated with cytotoxicity [26]. Moreover, our quantitative phytochemical study of MEMC demonstrated the presence of large quantities of flavonoids (Table 2), and the qualitative phytochemical screening revealed the existence of alkaloids, flavonoids, tannins, and steroids, which can also have cytotoxic effects.

The most common cause of death among individuals older than 60 years worldwide is ischemic stroke, often caused by the blockage of the cerebral artery due to embolus or local thrombus. Streptokinase was the first introduced clinical thrombolytic agent and has shown great efficacy but is also associated with limitations, including hemorrhagic complications due to the erosion of circulating fibrinogen and factors V and VII. Thrombolytic agents activate the plasminogen enzyme, which disrupts cross-linked fibrin meshes to produce soluble clots and initiates additional proteolysis activities that involve several enzymes to recover blood flow blocked by occlusions. Some new thrombolytic agents have recently been introduced for the treatment of myocardial infarction, deep vein thrombosis, thromboembolic strokes, and pulmonary embolism to clear obstructed arteries and eliminate the permanent injury of perfused tissues, such as the myocardium, leg muscles, and brain [27]. Our extract displayed moderate thrombolytic activity compared with that of the positive control, streptokinase, which may be due to the presence of bioactive secondary metabolites, such as alkaloids, tannins, and saponins, which were identified in the plant extract during the phytochemical study.

The denaturation of several proteins can occur due to inflammatory activities, particularly in conditions such as arthritis. Protection against protein denaturation is a key mechanism associated with NSAID use, which also have a suppressive effect against cyclooxygenase, which is thought to play a large role in the arthritic inflammation process [13]. Indomethacin and phenylbutazone are examples of conventional NSAIDs, which not only block the enzymatic activities of COX but also prevent protein denaturation. In our present study, anti-inflammatory activity was assessed using a protein denaturation protocol, in which denaturation was induced by heat treatment. MEMC was able to inhibit 46.66 ± 0.88% and 61.22 ± 0.87% of protein denaturation at concentrations of 250 and 500 µg/mL, respectively, compared with diclofenac sodium (the standard drug), which inhibited 83.3 ± 0.44% and 92.77 ± 0.34% of denaturation at 250 and 500 µg/mL concentrations, respectively. Compared with the standard drug, our plant extract presented minimum significant (*p* ˂ 0.001) activity. Secondary metabolites, including polyphenols and tannin, which were identified in the preliminary test, may be responsible for this anti-inflammatory activity, and *M. capitulata* is used in traditional medicinal regimens to treat rheumatic arthritis.

We analyzed the analgesic activity of MEMC by performing an acetic acid-induced abdominal writhing assay in a mouse model. After completing the acetic acid treatment, our tested mice presented with writhing behaviors due to abdominal constriction [28]. This assay reveals pain perception in response to the production of pro-inflammatory endogenous mediators, such as prostaglandins, histamines, serotonin, COX, lipoxygenase, and cytokines within the peripheral tissues and fluids, which impact prostaglandin stimulation between the peritoneal cavities and enhances inflammation by increasing capillary permeability. The agents that minimize writhing behaviors also reduce pain sensation and are commonly known as prostaglandin synthesis inhibitors or inhibitors of other inflammatory mediators [29,30]. In our assay, when MEMC was administered orally, reduced acetic acid-induced abdominal pain was observed, manifested as decreased writhing activity and indicating analgesic activity. These findings suggest that MEMC may regulate prostaglandin production, resulting in a significant analgesic outcome (Table 3). The formalin-induced paw-licking assay was also performed to investigate differences in the analgesic activity of MEMC mediated through central and peripheral mechanisms [31]. The formalin-induced paw-licking assay induces a sustained pain sensation and can be used to explore a biphasic nociceptive response (both early and late phases), which are mediated by independent mechanisms. The early preliminary phase (0–5 min) started when mice were injected intraperitoneally due to the gradual chemical stimulation of sensory C-fibers. Several types of opioid-based drugs are sensitive to intense neurogenic inflammation, and centrally acting anti-inflammatory drugs may suppress the ending phase (15–30 min), which is thought to cause an inflammatory response [32,33,34]. The formalin experiment revealed that MEMC treatment significantly diminished the formalin-induced pain reaction during both the early and late phases significantly, in a dose-dependent manner (Table 4), suggesting that the MEMC extract presents both central and peripheral analgesic effects.

During the drug discovery and development process, numerous molecular structures are appraised, and several parameters can be used to identify which chemicals to synthesize and test to select the most effective compounds that have potential therapeutic applications for patients. These molecules must exhibit biological activities, which can include toxicity. Selected bioactive compounds can be characterized by using an online tool (SwissADME), which is based on Lipinski’s rule of five and displays each compound’s drug-likeness, pharmacokinetics, and physicochemical features. Lipinski’s rule of five suggests that a compound or drug that is administered orally must remain within the limits of molecular weight (<500 amu), lipophilicity value (≤5), and hydrogen bond acceptor (<5) and donor (≤10) sites. Drugs and compounds that break these rules can be characterized by limited bioavailability. The ADME properties of our selected compounds are demonstrated in Table 5. Our outcomes revealed that all of the tested compounds maintained complied with Lipinski’s rule of five, suggesting that they are appropriate for further study. We also ascertained the toxicological features of our selected compounds using an online tool (admetSAR) to ensure safety. Toxicity is a vital parameter that must be evaluated for any effective drug candidate. The toxicological features of the examined compounds, including the Ames toxicity and carcinogenicity values, are shown in Table 6.

Molecular docking is an important computational strategy that is frequently used to predict ligand–target interactions to determine whether and how active compounds bind with key enzymes and can provide insights into the probable molecular mechanisms through which compounds exert pharmacological actions when combined with experimental studies. In our study, ten major *M. capitulata* compounds were selected: 3-(4-hydroxy-3 methoxyphenyl) acrylaldehyde, 4-hydroxybenzaldehyde, orcinol glucoside, pilosidine, capituloside, curcapital, crassifogenin C, breviscaside A, methyl-4-O-coumaroylquinate, and 2,6-dimethoxy-benzoic acid. These compounds were tested against four enzymes: 20YE, 1HD2, 1A5H, and 6COX. The obtained docking scores for all compound–enzyme pairs are presented in Table 7. The analgesic docking scores for 3-(4-hydroxy-3 methoxyphenyl)acrylaldehyde (−5.19), 4-hydroxybenzaldehyde (−5.41), curcapital (−5.32), breviscaside A (−5.25), and 2,6-dimethoxy-benzoic acid (−5.92) illustrated maximum binding affinities for the 2OYE enzyme. The antioxidant docking scores for 4-hydroxybenzaldehyde (−5.25), curcapital (−4.26), and methyl-4-O-coumaroylquinate (−4.59) showed significant binding scores for the 1HD2 enzyme, and 4-hydroxybenzaldehyde (−6.00), orcinol glucoside (−6.55), curcapital (−5.86), and 2,6-dimethoxy-benzoic acid (−5.62) showed the best binding affinities against 1A5H, displaying thrombolytic potential. The anti-inflammatory effects of 4-hydroxybenzaldehyde (−7.46), curcapital (−9.32), and crassifogenin C (−8.20) were suggested by the promising binding scores against 6COX. This study indicated that 4-hydroxybenzaldehyde, orcinol glucoside, curcapital, crassifogenin C, and 2,6-dimethoxy-benzoic acid could represent the most responsible bioactive components in MEMC, demonstrating analgesic, antioxidant, thrombolytic, and anti-inflammatory activity. These outcomes are supported by scientific reports that curcapital and 2,6-dimethoxy-benzoic acid have previously been demonstrated to display analgesic, antioxidant, and anti-inflammatory properties [6].

## 5. Conclusions

In summary, this study showed that MEMC has significant antioxidant, cytotoxic, thrombolytic, anti-inflammatory, and analgesic activities. The plant extract may have potential applications for the treatment of several chronic diseases, likely mediated by the phenols and flavonoids that are present in the plant extract, which can act independently or synergistically. Our computational study also revealed that 4-hydroxybenzaldehyde, orcinol glucoside, curcapital, crassifogenin C, and 2,6-dimethoxy-benzoic acid have good ADME/T properties and maximal binding affinities against the tested proteins. Overall, the characteristics of this plant extract suggest that MEMC may serve as a prospective candidate for establishing new drug compounds with numerous pharmacological targets. However, further extensive studies remain necessary to identify the secondary metabolites that are responsible for the observed biological activities to determine the underlying mechanism associated with these therapeutic activities.

## Figures and Tables

**Table 1:** Result of preliminary phytochemical analysis of methanol leaf extract of *Molineria capitulata* (MEMC).

Phytochemicals	MEMC
Alkaloids	+
Carbohydrates	+
Flavonoids	+
Phenols	+
Tannins	+
Saponins	−
Reducing sugar	+
Steroids	−
Glycosides	+
Terpenoids	+

(+) = present, and (−) = absent.

**Table 2:** Total phenolic and flavonoid contents of methanol leaf extract of *Molineria capitulata* (MEMC).

Tested Extract	Total Phenol Content (mg GA/g DE)	Total Flavonoid Content (mg QE/g DE)
MEMC	148.67 ± 1.15	24.00 ± 0.00

Each value in the table is expressed as mean ± SEM (*n* = 3). GAE, gallic acid equivalent; QE, quercetin equivalent.

**Table 3:** Analgesic effect of MEMC in acetic acid induced writhing test in mice.

Groups	Treatment	Dose (mg/kg)	Number of Writhing	Inhibition (%)
Control	1% Tween-80	0.1	61.4 ± 0.66	-
Positive Control	Diclofenac-Na	10	20.8 ± 0.75 ***	66.12
Plant Extract	MEMC	400	27.4 ± 0.9 ***	55.3
	MEMC	200	46.4 ± 1.3 ***	24.4

Values are presented as mean ± SEM (*n* = 5). *** *p* < 0.001 compared with the control group (Dunnett’s test). MEMC, methanol leaf extract of Molineria capitulata.

**Table 4:** Analgesic effect of MEMC in formalin induced paw licking test in mice.

Treatment (mg/kg)	Licking Time (sec) (Mean ± SEM)			
	Early Phase (0–5 min)	Inhibition (%)	Late Phase (15–30 min)	Inhibition (%)
Control (0.1 mL/mouse)	30.60 ± 5.3	-	44.44 ± 0.65	-
Diclofenac-Na (10)	17.40 ± 0.35 ***	75.69	16.80 ± 0.75 ***	62.16
MEMC (400)	34.60 ± 0.68 ***	51.67	20.60 ± 0.87 ***	53.60
MEMC (200)	56.00 ± 07.8 ***	21.78	33.60 ± 1.55 ***	24.32

Values are presented as mean ± SEM (*n* = 5). *** *p* < 0.001 compared with the control group (Dunnett’s test). MEMC, methanol leaf extract of *Molineria capitulate*; SEM, Standard Error Mean.

**Table 5:** ADME property prediction for the major selected bioactive compounds of *M. capitulata*, obtained using Swiss ADME online tool [17].

Compounds	MW ^1^	HBA ^2^	HBD ^3^	Log P ^4^	MR ^5^	Rule of Five ^6^
3-(4-hydroxy-3-methoxyphenyl)acrylaldehyde	178.18	3	1	1.56	50.06	0
4-hydroxybenzaldehyde	122.12	2	1	1.17	33.85	0
Orcinol glucoside	286.28	7	5	−0.47	67.58	0
Pilosidine	478.45	11	7	−0.10	114.99	2
Capituloside	478.45	11	7	−0.10	114.99	2
Curcapital	310.26	6	4	1.97	84.71	0
Crassifogenin C	348.30	8	6	0.54	84.81	1
Breviscaside A	480.46	11	8	−0.10	115.61	2
Methyl-4-O-coumaroylquinate	352.34	8	4	0.42	85.80	0
2,6-dimeyhoxy-benzoic acid	182.17	4	1	1.18	46.39	0

^1^ MW: Molecular weight-(acceptable range: <500 g/mol); ^2^ Hydrogen bond acceptor (HBA)-(Acceptable range: ≤10); ^3^ Hydrogen bond donor (HBD) (Acceptable range: ≤5); ^4^ Lipophilicity (Log *P*_o/w_) (acceptable range < 5); ^5^ Molar refractivity (MR) should be between 40 and 130. ^6^ Rule of five: Number of violations of Lipinski’s rule of five: accepted range: 0–4.

**Table 6:** Toxicological property predications of the major selected bioactive compounds of *M. capitulata* using admetSAR online server.

Compounds	Parameters	
	AMES Toxicity	Carcinogens
3-(4-hydroxy-3-methoxyphenyl)acrylaldehyde	Non AMES toxic	Non-Carcinogens
4-hydroxybenzaldehyde	Non AMES toxic	Non-Carcinogens
Orcinol glucoside	AMES toxic	Non-Carcinogens
Pilosidine	Non AMES toxic	Non-Carcinogens
Capituloside	Non AMES toxic	Non-Carcinogens
Curcapital	AMES toxic	Non-Carcinogens
Crassifogenin C	Non AMES toxic	Non-Carcinogens
Breviscaside A	Non AMES toxic	Non-Carcinogens
Methyl-4-O-coumaroylquinate	Non AMES toxic	Non-Carcinogens
2,6-dimeyhoxy-benzoic acid	Non AMES toxic	Non-Carcinogens

**Table 7:** Docking scores of the major selected bioactive compounds of *M. capitulata.*

Compounds	Docking Scores (kcal/mol)			
	1HD2	1A5H	20YE	6COX
3-(4-hydroxy-3-methoxyphenyl)acrylaldehyde	-	−2.02	−5.19	−6.26
4-hydroxybenzaldehyde	−5.25	−6.00	−5.41	−7.46
Orcinol glucoside	−3.54	−6.55	−4.16	−5.27
Pilosidine	−3.27	−3.95	−4.44	-
Capituloside	−3.27	−3.96	−4.69	-
Curcapital	−4.26	−5.86	−5.32	−9.32
Crassifogenin C	−2.86	−4.61	−4.86	−8.20
Breviscaside A	−2.69	−4.29	−5.25	-
Methyl-4-O-coumaroylquinate	−4.59	−4.75	−3.87	−5.91
2,6-dimeyhoxy-benzoic acid	−3.69	−5.62	−5.92	−7.11
Standard drug (Ascorbic acid/Streptikinsae/Diclofenac-Na)	−5.13	−4.53	−4.59	−7.26

## Data Availability

Available data are presented in the manuscript.

## Abbreviations

MEMC: methanol extract of *M. capitulata* leaves; FCR: Folin-Ciocalteau reagent; UV: Ultra-violet; BW: Body weight; TPC: Total phenol content; TFC: Total flavonoid content; EPM: elevated plus maze; TST: tail suspension test; SEM: Standard error mean; p.o.: Per oral; i.p.: Intraperitoneal.
